# Attenuation of the association between sugar-sweetened beverages and diabetes risk by adiposity adjustment: a secondary analysis of national health survey data

**DOI:** 10.1007/s00394-018-1716-z

**Published:** 2018-05-15

**Authors:** Yi Jing, Thang S. Han, Majid M. Alkhalaf, Michael E. J. Lean

**Affiliations:** 1 Human Nutrition, School of Medicine, University of Glasgow, Level 2, New Lister Building, 10-16 Alexandra Parade, Royal Infirmary, Glasgow, G31 2ER Scotland UK; 2 Institute of Cardiovascular Research, Royal Holloway, University of London, Egham, UK; 3 Department of Endocrinology, Ashford and St Peter’s NHS Foundation Trust, Chertsey, UK

**Keywords:** BMI, Obesity, Health survey, HbA_1c_, Nutrition, Sucrose

## Abstract

**Purpose:**

While weight gain and obesity are the dominant factors, dietary sugar and specifically sugar-sweetened beverages (SSB) has been implicated in causing type 2 diabetes (T2DM). We assessed how much of the apparent effect of SSB is explained by adiposity, but not captured by adjustment for BMI, which is a poor index of body fat.

**Methods:**

We examined data from 5187 adults (mean age 50.8 years, SD = 16.4, 172 (3.3%) T2DM), from the Scottish Health Survey 2003 and 2008–2010 databases. Logistic regression was used to assess the association between SSB consumption and T2DM (non-insulin treated) and its attenuation (reduction in odds ratios, ORs), after entering published anthropometric indices of adiposity into the regression model, adjusted for age, sex, social class, education, smoking, alcohol consumption and physical activity.

**Results:**

Compared with low SSB categories (“less often/never”, once/week or 1–3 times/month), the OR without adiposity adjustment for having T2DM in high SSB consumers (2–3, 4–5, ≥ 6/day) was 2.56 (95% CI 1.12–5.83; *p* = 0.026). That OR was marginally changed by adjusting for BMI (+ 4.3%), WC (+ 5.5%) or total body fat (− 4.3%), but greatly attenuated by adjusting for estimated %body fat (− 23.4%). These indices had similar influences on the associations between SSB and T2DM combining known T2DM patients with unknown HbA_1c_ > 6.5%, > 48 mmol/mol.

**Conclusions:**

Associations between SSB and T2DM are attenuated more markedly by adjustment with estimated %body fat than with BMI, indicating an adiposity effect not captured using BMI. Future research should employ best available estimates of adiposity.

## Introduction

The global prevalence of adult type 2 diabetes mellitus (T2DM) has nearly doubled from 4.7% in 1980 to 8.5% (422 million cases) in 2014, coinciding with trends in obesity and overweight [1]. The burden of diabetes was found strongly attributable to overweight and physical inactivity by comparative risk assessment [2]. Furthermore, T2DM has now been proven to be preventable, with body weight control being the dominant factor, with lesser influences from physical activity, and more minor dietary factors such as reducing saturated fats, increasing fruit and vegetables, magnesium and dietary fibre [3–5].

For historical reasons and through limited understanding of nutrition, a direct association has been assumed by many between raised ‘blood sugar’ and dietary sugar [5]. Ecological data support an association (not necessarily causal) between average sugar use and diabetes prevalence [6], but the relationship is potentially confounded by more frequent overweight and obesity in countries with more diabetes: attempting to correct this for average BMI will not remove the influence of fatness, as BMI correlates rather weakly with body fat [7]. Obtaining reliable quantitative data on total dietary intake of individuals, from which to estimate sugar consumption, is not possible because overweight and obese individuals (who must have relatively high energy consumptions to remain overweight) are well known to under-report total food consumptions systematically [8, 9]. However, reported consumptions of specific foods and drinks, such as sweetened beverages, are considered more reliable because their consumption patterns are more stable [10]. Sugar-sweetened beverages (SSB) have come under particular scrutiny in relation to diabetes because they provoke a high blood glucose peak, and because calories in liquid have a weaker effect on satiety than in solid form [11].

A large recent study by Imamura et al. found BMI marginally attenuates the association between SSB consumption and T2DM [12], but neither this nor any previous population-based studies have investigated measurements or estimates of body fat itself as a confounding factor which might be responsible for this association. The present study was conducted to establish the extent to which the association between SSB intake and T2DM is attenuated by commonly used indices of adiposity including BMI, waist circumference (WC), and estimates of total adipose tissue mass (TATM) and percent body fat (BF%).

## Methods

### Data source

This study combined available datasets of Scottish Health Surveys 2003, 2008, 2009 and 2010 [13] and used ‘complete cases’ of individuals for whom the necessary data were collected to be able to compare all the adiposity indices. The whole survey program comprised a household survey and an individual survey, among a total of 38,863 free-living participants elected from electoral registers to be nationally representative. Those under 18 years old at last birthday, pregnant, and on insulin treatment (potentially cases of T1DM, or T2DM with secondary weight gain) were all excluded (*n* = 10,734) leaving 28,136 participants. For 6358 of these participants after exclusions, nurse-led interviews were conducted using standard questionnaires to collect information on the participants’ sociodemographic characteristics and lifestyle variables, providing measured anthropometric data and blood samples for glycated haemoglobin (HbA_1c_).

Physical activity level was assessed from reported number of days per week with any physical activities lasting ≥ 30 or 10–29 min of exercise sessions and categorised into groups of none (physical inactivity), active < 1 day/week, 1 or 2 days/week, 3 or 4 days/week and ≥ 5 days/week. Smoking status was defined as never smoker, used to smoke occasionally, used to smoke regularly and current smoker. Alcohol consumption was defined according to frequency of drinking: never-drinkers, drinking once or twice/year, once every couple of months, once or twice a month, once or twice a week, 3 or 4 times/week, 5 or 6 times/week and almost every day. Education level attainment was grouped into other school level or no qualifications, standard grade or equivalent, higher grade or equivalent, higher national certificate/diploma or equivalent and degree or higher. Socioeconomic classes were determined from occupation and classified as unskilled manual, semi-skilled manual, skilled manual, skilled non-manual, managerial technical and professional [13].

### Sugar-sweetened beverage intakes

During interviews, SSB consumption frequency (without volume) of soft drinks was recorded as “less often or never”, 1–3 times per month, 1, 2–4, 5–6 times per week, 1, 2–3, 4–5, ≥ 6, times per day, which were categorised from 1 to 9 respectively for analysis.

### Diabetes cases and HbA_1c_

A previous diagnosis of diabetes by doctors was recorded. Among those without previous diagnosis of T2DM, those who had HbA_1c_ > 6.5% (48 mmol/mol) were added to the diagnosed T2DM group, in a second model.

### Indices of adiposity

Four established indices of adiposity were assessed, to test their effect on the association between SSB and diabetes: BMI (kg/m^2^), WC (cm), total adipose tissue mass [TATM (kg)] calculated using equation derived by Al-Gindan et al. [14] which contains body weight and height for both sexes, plus WC for male and age for female, and BF% (% of body weight) using equation derived by Lean et al. [7] which contains WC and age for both sexes.

### Statistical analysis

From 28,136 participants we extracted 5187 cases with complete data for all variables of interest, (i.e. 22,949 with incomplete data or did not meet inclusion criteria were excluded). There were no important differences between the extracted and excluded groups in gender distribution (M/F = 54.8/45.2 vs. 55.5/44.5%. *p* = 0.176), mean age (50.8 vs. 50.3 years, *p* = 0.093) or BMI (27.5 vs. 27.8 kg/m^2^, *p* < 0.05). Predictor variables were categorised into three categories: For SSB, category 1–3 indicates lowest drinking frequency of “less often or never”, 1–3 times/month or once/week; category 4–6 indicates drinking frequency of 2–4 or 5–6 times/week or once/day, and category 7–9 indicates highest drinking frequency of 2–3, 4–5 or ≥ 6/day. Chi-square test was used to assess differences in T2DM prevalence across different categories of SSB and adiposity and logistic regression to assess the association between SSB and previously diagnosed T2DM. Indices of adiposity were individually entered into the logistic regression model to examine the degree in which this association was affected, indicated by changes in odds ratios (ORs). Analysis was repeated with the addition of cases without previous diagnosis of T2DM but with HbA_1c_ > 6.5% (48 mmol/mol). Data were adjusted for age, sex, social class, smoking status, alcohol consumption, education attainment and physical activity level. Analyses were performed using SPSS V.22.0 (SPSS Inc, Chicago, IL, USA). Statistical significance was accepted when *p* < 0.05.

The influences of adiposity on the association between SSB and T2DM were expressed as percentage reduction from 100%, by dividing the “difference between OR after adjustment minus OR before adjustment for adiposity” over “OR before adjustment for adiposity”.

## Results

Men and women had similar mean age (51.1 and 50.5 years), BMI (27.6 and 27.4 kg/m^2^) and HbA_1c_ 5.32% (34.7 mmol/mol) and 5.30% (34.4 mmol/mol). Men had larger WC than women (by 10 cm, *p* < 0.001) while women had greater amount of TATM (4.7 kg, *p* < 0.001) and BF% (11.6% of body weight, *p* < 0.001) than men (Table 1).

**Table 1:** Subject characteristics including age, anthropometry, estimated skeletal and fat masses and percentages and HbA_1c_

	Men (*n* = 2347)		Women (*n* = 2840)		Independent *t* test for group differences (male values minus female values)		
	Mean	SD	Mean	SD	Mean	95% CI	*p*
Age (years)	51.1	16.6	50.5	16.3	0.6	− 0.3, 1.5	0.228
Weight (kg)	83.6	14.5	70.6	14.3	13.0	12.2, 13.7	< 0.001
Height (cm)	174.0	7.1	160.6	6.7	13.4	13.0, 13.8	< 0.001
BMI (kg/m^2^)	27.6	4.5	27.4	5.4	0.2	− 0.1, 0.5	0.139
Waist (cm)	97.1	11.9	87.1	13.0	10.0	9.3, 10.7	< 0.001
TATM (kg)	24.6	8.1	29.3	11.0	− 4.7	− 5.2, − 4.1	< 0.001
BF% (% body weight)	28.4	7.4	40.0	7.4	− 11.6	− 12.0, − 11.2	< 0.001
HbA_1c_ (%)^a^	5.32	0.49	5.30	0.47	0.02	− 0.01, 0.05	0.153
HbA_1c_ (mmol/mol)^a^	34.7	5.3	34.4	5.2	0.2	− 0.1, 0.6	0.153

^a^HbA_1c_ in 1823 men and 2086 women who were not previously diagnosed with T2DM

The prevalence of T2DM was 1.8% in men who had the lowest intake of SSB (“less often or never”, once/week or 1–3 times/month), 1.3% in men who consumed between 2 and 4 or 5–6 times/week and once/day, and 4.9% in men who had the highest intake of SSB (2–3, 4–5, ≥ 6 per day) (*χ*^2^ = 19.0, *p* < 0.001); the respective values of T2DM prevalence across SSB categories for women were 1.7, 2.7 and 3.3% (*χ*^2^ = 2.8, *p* < 0.242). A significantly greater prevalence of T2DM was observed for those in higher tertiles of all adiposity measures in both sexes (Table 2).

**Table 2:** Distribution of SSB, indices of adiposity and lifestyle factors in men and in women in patients with diagnosed T2DM and those with no previous T2DM diagnosis but with HbA_1c_ > 6.5% (48 mmol/mol)

	Men						Women					
	Diagnosed T2DM (*n* = 85 cases and 2262 non-cases)			Diagnosed T2DM + no previous T2DM diagnosis with HbA_1c_ > 6.5% (48 mmol/mol) (*n* = 114 cases and 2233 non-cases)			Diagnosed T2DM (*n* = 87 cases and 2753 non-cases)			Diagnosed T2DM + no previous T2DM diagnosis with HbA_1c_ > 6.5% (48 mmol/mol) (*n* = 116 cases and 2724 non-cases)		
Categories of SSB intake and indices of adiposity^a^	%	*χ* ^2^	*p*	%	*χ* ^2^	*p*	%	*χ* ^2^	*p*	%	*χ* ^2^	*p*
SSB category 1–3	1.8	19.0	< 0.001	3.2	12.8	0.002	1.1	2.8	0.242	1.7	2.9	0.236
SSB category 4–6	1.3			2.7			2.7			4.6		
SSB category 7–9	4.9			6.1			3.3			4.2		
BMI lowest tertile (M < 25.48, F < 24.47 kg/m^2^)	1.3	40.3	< 0.001	1.9	46.8	< 0.001	0.7	63.4	< 0.001	1.2	81.0	< 0.001
BMI middle tertile (M ≥ 25.48-, F ≥ 24.47 kg/m^2^)	2.6			3.6			1.8			2.3		
BMI highest tertile (M ≥ 29.00, F ≥ 28.88 kg/m^2^)	7.0			9.0			6.7			8.8		
WC lowest tertile (M < 91.25, F < 80.55 cm)	1.4	42.6	< 0.001	1.9	47.1		0.5	53.5	< 0.001	0.7	80.9	< 0.001
WC middle tertile (M ≥ 91.25, F ≥ 80.55 cm)	2.3			3.6			2.4			2.8		
WC highest tertile (M ≥ 101.70, F ≥ 91.15 cm)	7.1			9.0			6.2			8.6		
TATM lowest tertile (M < 26.52, F < 37.01 kg)	0.7	48.9	< 0.001	1.3	62.4	< 0.001	1.3	40.3	< 0.001	1.3	43.4	< 0.001
TATM middle tertile (M ≥ 26.52, F ≥ 37.01 kg)	2.3			2.9			2.6			2.4		
TATM highest tertile (M ≥ 31.44, F ≥ 43 kg)	6.1			8.1			7.0			7.2		
BF% lowest tertile (M < 24.93, F < 36.37% of weight)	1.0	50.6	< 0.001	1.5	56.0		0.2	81.8	< 0.001	0.3	119.6	< 0.001
BF% middle tertile (M ≥ 24.93-, F ≥ 36.37- % of weight)	2.4			3.4			1.9			2.2		
BF% highest tertile (M ≥ 31.36, F ≥ 43.14% of weight)	7.4			9.6			7.1			9.7		

^a^For SSB, category 1–3 indicates lowest drinking frequency of “less often or never”, 1–3 times per month or 1 time per week; category 4–6 indicates drinking frequency of 2–4, 5–6 times per week or 1 time per day, and category 7–9 indicates highest drinking frequency of 2–3, 4–5 or ≥ 6 per day. For indices of adiposity, three sex-specific tertiles were created

We found similar influences by these indices on the association between SSB and known T2DM patients combined with unknown T2DM who had HbA_1c_ > 6.5% (48 mmol/mol).

**Fig. 1:**
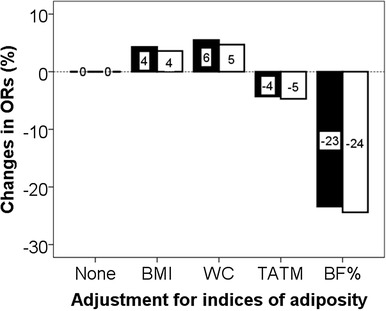
Attenuation of the association between SSB and T2DM (solid bars) and between SSB and previously diagnosed T2DM + no previous T2DM diagnosis with HbA_1c_ > 6.5% (48 mmol/mol) (open bars) by different indices of adiposity indicated by the relative reduction of ORs

Table 3 shows that compared with those with the lowest SSB intake (“less often or never”, once/week or 1–3 times/month), OR for having T2DM in those with highest SSB intake (2–3, 4–5, ≥ 6/day), without adiposity adjustment, was 2.56 (95% CI 1.12–5.83, *p* = 0.026). That OR was minimally changed by adjustment for BMI (+ 4.3%), WC (+ 5.5%) or total body fat (− 4.3%), while greatly attenuated by %body fat (− 23.4%) (Fig. 1). There was a similar association between SSB and T2DM in the larger combined model, including known (diagnosed) (*n* = 172) with those previously undiagnosed but with HbA_1c_ > 6.5% (48 mmol/mol) (*n* = 230): OR = 1.93 (95%CI: 1.01–3.69, *p* = 0.046) (Table 4) and similar magnitude of changes in this association by indices of adiposity (Fig. 1).

**Table 3:** Logistic regression analysis to assess relative changes in the association of SBB and previously diagnosed T2DM by indices of adiposity

Predictor variables^a^	Diagnosed T2DM (*n* = 172 cases and 5015 non-cases)			
	OR	95% CI	*p*	Relative changes in OR by indices of adiposity^b^ (%)
SSB 1–3 (referent)	1	–	–	–
SSB 4–6	1.19	0.48–2.96	0.707	–
SSB 7–9	2.56	1.12–5.83	0.026	0
SSB 1–3 + BMI (referent)	1	–	–	–
SSB 4–6 + BMI	1.28	0.51–3.22	0.594	–
SSB 7–9 + BMI	2.67	1.16–6.14	0.021	+ 4.3
SSB 1–3 + WC (referent)	1	–	–	–
SSB 4–6 + WC	1.21	0.48–3.04	0.683	–
SSB 7–9 + WC	2.70	1.07–5.60	0.020	+ 5.5
SSB 1–3 + TATM (referent)	1	–	–	–
SSB 4–6 + TATM	1.25	0.50–3.12	0.639	–
SSB 7–9 + TATM	2.45	1.07–5.63	0.035	− 4.3
SSB 1–3 + BF% (referent)	1	–	–	–
SSB 4–6 + BF%	1.07	0.43–2.68	0.885	–
SSB 7–9 + BF%	1.96	0.85–4.49	0.114	− 23.4

All models were adjusted for age, sex, social class, education attainment, smoking status, alcohol consumption and physical activity level

^a^For SSB, category 1–3 indicates lowest = “less often/never”, 1–3 times/month or once/week; category 4–6 indicates intermediate = 2–4 or 5–6 times/week or once/day, and category 7–9 indicates highest intake = 2–3, 4–5 or ≥ 6/day. Indices of adiposity and age were entered as continuous variables

^b^Relative changes in OR by indices of adiposity were calculated by dividing (the difference between OR after adiposity adjustment and OR before adiposity adjustment) over OR before adiposity adjustment × 100%, e.g. to calculate changes by BF%: [(1.96–2.56)/2.56] × 100% = − 23.4%

**Table 4:** Logistic regression analysis to assess changes in the association of SBB and previously diagnosed T2DM + no previous T2DM diagnosis with HbA_1c_ > 6.5% (48 mmol/mol) by indices of adiposity

Predictor variables^a^	Diagnosed T2DM + no previous T2DM diagnosis with HbA_1c_ > 6.5% (48 mmol/mol) (230 cases and 4957 non-cases)			
	OR	95% CI	*p*	Relative changes in OR by indices of adiposity^b^ (%)
SSB 1–3 (referent)	1	–	–	–
SSB 4–6	1.34	0.66–2.70	0.416	–
SSB 7–9	1.93	1.01–3.69	0.046	0
SSB 1–3 + BMI (referent)	1	–	–	–
SSB 4–6 + BMI	1.44	0.71–2.94	0.314	–
SSB 7–9 + BMI	2.00	1.04–3.87	0.038	+ 3.6
SSB 1–3 + WC (referent)	1	–	–	–
SSB 4–6 + WC	1.36	0.67–2.77	0.398	–
SSB 7–9 + WC	2.02	1.05–3.90	0.035	+ 4.7
SSB 1–3 + TATM (referent)	1	–	–	–
SSB 4–6 + TATM	1.40	0.69–2.85	0.354	–
SSB 7–9 + TATM	1.84	0.96–3.54	0.069	− 4.7
SSB 1–3 + BF% (referent)	1	–	–	–
SSB 4–6 + BF%	1.20	0.59–2.45	0.611	–
SSB 7–9 + BF%	1.46	0.76–2.82	0.258	− 24.4

All models were adjusted for age, sex, social class, education attainment, smoking status, alcohol consumption and physical activity level

^a^For SSB, category 1–3 indicates lowest = “less often/never”, 1–3 times/month or once/week; category 4–6 indicates intermediate = 2–4 or 5–6 times/week or once/day, and category 7–9 indicates highest intake = 2–3, 4–5 or ≥ 6/day. Indices of adiposity and age were entered as continuous variables

^b^For calculations of attenuation, see footnote in Table 3

We observed that BF% had stronger associations than BMI with SSB (*χ*^2^ = 27.0, *p* < 0.001 vs. 21.2; *p* < 0.001) and with T2DM (*χ*^2^ = 130.2; *p* < 0.001 vs. 102.5; *p* < 0.001). Among T2DM, we found that, compared with patients with HbA_1c_ < 6.5% (48 mmol/mol), more of those with HbA_1c_ > 6.5% (48 mmol/mol) had figures in the highest tertile of WC (26.6 vs. 73.4%, *χ*^2^ = 8.2, *p* = 0.016) and BF% (25.6 vs. 74.4%, *χ*^2^ = 12.3, *p* = 0.002) but did not have significantly higher BMI (*p* = 0.253), TATM (*p* = 0.171) or SSB intake (*p* = 0.124).

We examined relationships between SSBs and HbA_1c_ in people who were not diabetic: the associations were trivial though statistically significant, and no effect was evident from adjustment for BMI, WC or BF%. We also examined separately those who were not previously diagnosed with diabetes but had HbA_1c_ levels > 6.5% (> 48 mmol/mol). Numbers with complete data in this interesting group were too small for separate analysis.

## Discussion

The present study shows that the weak association of SSB and T2DM, found fairly consistently in epidemiological studies, was changed marginally by BMI and WC but more markedly by better indices of body fat. Body fatness is, therefore important confounding factor, reflecting the complex interactions of SSB and caloric intake in the development of obesity and T2DM.

SSB consumption has previously been related to high BMI [15], particularly among adults aged under 45 years [16], which is supported by our results. Imamura et al. [12] conducted a meta-analysis of 17 studies comprising 38,253 cases with T2DM studied over 10,126,754 person years, mostly using food frequency questionnaires for dietary assessment. The positive association between SSB consumption and risk of T2DM was attenuated by BMI, indicated by a small reduction in relative risk from 1.18 (95% CI 1.09–1.28) to 1.13 (95% CI 1.06–1.21). Using our calculation method, this reduction represents 4.2% attenuation in the association between SSB and T2DM. Our present study found a little change after adjustment by BMI, WC (+ 4–6%) or estimated TATM (− 4%), but substantially greater attenuation by estimated BF% (-23%).

BMI is frequently used as an indicator of adiposity and routinely recorded in large epidemiological studies [17]. However, BMI has only weak correlation with body fat, because BMI does not distinguish between body fat and muscle mass [18]. Thus, BMI explains only 60–70% of variance in percent body fat measured by reference techniques such as underwater weighing [7]. The present study found that BMI explained 72.4% of variance of BF% as estimated by previous published equations [7], therefore, about 30% of residual variance from body fat remains to confound analyses. In slimmer, more muscular and younger populations, the relationship between BMI and body fat may be even weaker. WC, now also available in most health surveys, has a slightly better correlation with total body fat, as well as indicating body fat distribution [19]. We found both BMI and WC had minimal effect on the association between SSB and T2DM. We also examined BF%, estimated by equations based again on simple measurements made in most health surveys [7, 14]. This provided the greatest attenuation, but these equations still only explain some 80% of variance in body fat, so the weakened remaining association between SSB consumption and T2DM includes substantial residual variance from body fat. The new estimates of body fatness using validated equations are preferable to BMI for other purposes.

The association of SSBs with health problems such as T2DM may be better explained by the modern patterns of eating, and snacking, which are characterised by high consumption of SSBs, than by the sugar itself. The current study is in line with this view. For decades, some have asserted fervently that sugar causes obesity and T2DM while others blamed artificial sweeteners for obesity and cancers without solid scientific evidence. During human evolution, the sweetest food we ever met—except honey—was human milk until the nineteenth century when unnaturally sweet drinks and sugary-fatty snacks were invented which had become abundant by the twentieth century. This sudden availability led to adverse effects, just as experimental animals fed “cafeteria diets” develop obesity and diabetes. Although glucose is an essential nutrient necessary for the function of every cell in our bodies, it is technically not essential in our diets, being readily generated from other foods. There are health benefits from sugar-containing fruits and vegetables, but sugar added during manufacturing confers no physical benefits. Added sugars have presented a reasonable target for taxation to raise revenue since the 1930s [20, 21], now proposed explicitly against obesity [22].

The term “added sugars” can be confusing. They include “refined sugars” (either natural, from cane, or synthetic, made from grain or beet crops), but sweetness is also added to foods from natural sugar, from raisins, apple juice, honey, etc. All are biologically identical once inside the gut. Sucrose (table sugar) is a disaccharide comprising equal parts glucose and fructose. The fructose molecule is converted into glucose, our primary cellular nutrient. The evidence linking total, or added, sugar consumption with poor health is weak. Consumption of SSBs is more reliably estimated and so has a stronger evidence base for associations with poor health. SSBs contribute about 13% of total sugar intakes in the United Kingdom, up to 30% in children, but still below 5% of calories [23]. Reducing sugar in SSBs from 10 to 8% would affect about 0.4% of total calories, and under 1% even for the heaviest users. There are theoretical small benefits from sugar reduction, especially for those with very high intakes namely reducing blood pressure [24] and cardiovascular diseases [25]. If cutting down sugar intake can reduce weight gains by 2–3 kg [26], that would definitely help delay the onset of weight-related diseases like T2DM. There is no evidence, however, to support recommending a lower upper limit of sugar intake of a relatively generous 10% of calories. Systematic reviews and meta-analyses find no weight-independent effect of sugar on diabetes development [5, 26].

Two much-disputed bodies of evidence indicate that problems with obesity lie not with sugar or with sweeteners themselves, but with the modern eating patterns of snacking or grazing, which are marked by high consumption of both very sweet drinks and sugary/fatty snacks. First, a meta-analysis of randomized controlled and prospective cohort studies found absolutely no effect on body weight when sugar is replaced with the same calories from other carbohydrates [26]. Second, although evidence is conflicting [27], artificially sweetened drinks are also associated with weight gain [28]. It must be emphasised that this is only an association, but experimental animals only gain weight when both drinking water and foods are sweetened, either with sugar or with artificial sweeteners [29]. Human epidemiology can be explained by essentially the same story: both SSBs and artificially sweetened “diet” versions cause tolerance to unnatural sweetness, which promotes weight gain, mainly by promoting consumption of very sweet, energy-dense foods [5, 29]. “Reverse causality” may contribute; people with weight problems tend to choose diet drinks, but obese people prefer sweeter tastes and sweet, high-fat foods [30], and children who eat more fast foods prefer sweeter tastes [31].

The strengths of the present study lie in its relatively large homogeneous sample, with a wide range of SSB intakes and adiposity which enables us to reliably assess their relationships with T2DM, but the study has limitations. Causal links between SSB and T2DM cannot be established in this cross-sectional study, and there is some uncertainty the degree to which, under advice after diagnosis of T2DM, patients may have modified their ‘diabetogenic’ dietary habits [32, 33], particularly among ethnic minority groups [34]. In keeping with other published studies, some other factors were not available that could affect the association of SSB consumption and diabetes, such as dietary fat and fibre intake. The data available did not provide reliable quantitative assessment of dietary components, and estimation of total energy intake was not possible with the data collected. However, SSB consumption frequency has been assumed by most researchers to relate indirectly with quantitative intake, and has consistently been shown to associate with diabetes. In our study, indices of adiposity were derived from validated anthropometric measurements, rather than measured directly. This is in common with all large epidemiological studies, since these indices provide both practical and economical approach to assessment of adiposity; “direct” methods of measuring adiposity such as magnetic resonance imaging are too expensive and time consuming to both investigators and participants, and there is no evidence that other indirect methods such as bioimpedance are better than anthropometric estimates [19].

In conclusion, the association between SSB and T2DM is  changed by adiposity, most markedly by %body fat, indicating the importance of adjustment for body fatness using validated equations such as those published by Lean et al. [7] and Al-Gindan et al. [14], rather than just BMI, in future research in this area.
